# Supplementary material for: Counteracting Roles of Lipidic Aldehydes and Phenolic Antioxidants on Soy Protein Oxidation Defined by a Chemometric Survey of Solvent and Mechanically Extracted Soybean Meals
Source: Antioxidants (Basel). 2023 Jul 13;12(7):1419. doi: 10.3390/antiox12071419 (PMC10376880; doi:10.3390/antiox12071419)
Supplement: Supplementary file 1 [file antioxidants-12-01419-s001.zip › antioxidants-2489358-supplementary.pdf]

Counteracting Roles of Lipidic Aldehydes and Phenolic Antioxidants on Soy Protein Oxidation Defined by a Chemometric Survey of Solvent and Mechanically Extracted Soybean Meals

Junwei Zhang [1], Pedro E. Urriola [2], Seth L. Naeve [3], Gerald C. Shurson [2,*] and Chi Chen [1,*]

[1] Department of Food Science and Nutrition, University of Minnesota, St. Paul, MN 55108, USA
[2] Department of Animal Science, University of Minnesota, St. Paul, MN 55108, USA
[3] Department of Agronomy and Plant Genetics, University of Minnesota, St. Paul, MN 55108, USA
* Correspondence: shurs001@umn.edu, chichen@umn.edu

Supplementary Data

Table S1. Sources of Chemicals and Reagents Used in Chemical Analysis, LC-MS Analysis, Structural Confirmation and Quantification.

Chemicals and reagents	Vendors
6-Hydroxy-2,5,7,8-tetramethylchroman-2-carboxylic acid (Trolox), Trichloroacetic acid	Acros Organics (Morris Plains, NJ)
Guanidine hydrochloride, 2-hydrazinoquinoline (HQ), Triphenylphosphine (TPP)	Alfa Aesar (Tewksbury, MA)
Methanol (HPLC grade)	Avantor performance materials (Radnor, PA)
Aldehyde standards*: 2-decenal, 2-hexenal, 2-heptenal, 2,4-heptadienal, octanal, 2-octenal, nonanal, 2-nonenal, 2,4-nonadienal	Bedoukian Research (Danbury, CT)
(2,2'-azino-bis (3-ethylbenzothiazoline-6-sulphonic acid) diammonium salt (ABTS)	Chem-Impex International (Wood Dale, IL)
Acetonitrile (HPLC-MS grade), Ammonium acetate (HPLC-MS grade), *p*-anisidine, 2,4-dinitrophenylhydrazone (DNPH), Formic acid (HPLC-MS grade), Hydrochloric acid, Isooctane (HPLC grade), Sulfadimethoxine (SDM), Water (HPLC-MS grade)	Fisher Scientific (Houston, TX)
Isoflavone standards#: Daidzein, Daidzin, Genistein, Genistin	MedChemExpress (Princeton, NJ)
2-2´-Dipyridyl disulfide (DPDS)	MP Biomedicals, LLC (Irvine, CA)
Tripentadecanoin (TG, 15:0, 15:0, 15:0)	Nu-Chek Prep, Inc (Elysian, MN)
Dimethyl sulfoxide (DMSO), Ethanol, Ethyl acetate, Folin–Ciocalteu reagent, Sodium carbonate, α-tocopherol^, γ-tocopherol^	Sigma-Aldrich (St. Louis, MO)

* Aldehyde standards were dissolved in methanol containing 10 μM acetone-d6 (internal standard) to obtain working standard solutions with the concentration of 0, 5, 12.5, 25, 50, 125, 250 and 500 μM.

# Isoflavone standards were firstly dissolved in DMSO to prepare stock solution (10 mg/mL), and then diluted with methanol containing 1 μM SDM (internal standard) to obtain working standard solutions with the concentration of 0, 0.125, 0.25, 0.5, 1.25, 2.5, 6.25, 12.5, 25 and 62.5 μg/mL.

^ Tocopherol standards were dissolved in methanol containing 5 μg/mL TG (internal standard) and then diluted to obtain working standard solutions with the concentration of 0, 0.05, 0.125, 0.25, 0.5, 1.25, 2.5, 5 and 12.5 μg/mL.

Table S2. AOAC (2007) Methods for the Proximate Analysis of SSBM and MSBM Samples

Proximate analysis components	Procedures
Crude protein	AOAC 984.13
Ash	AOAC 942.05
Ether extract	AOAC 920.39
Crude fiber	AOAC 978.10
Moisture	AOAC 934.01

Table S3. LC-MS Data Acquisition Conditions in a 10-minute Run.

Target compounds	Column type	Mobile phase	MS detection mode	Capillary and cone voltage	Source and desolvation temperature	Cone and desolvation gas	Collision gas
Aldehydes	BEH C18	A: H_2O containing 0.05% acetic acid (v/v) and 2 mM ammonium acetate<br>B: H_2O/ACN = 5:95 (v/v) containing 0.05% acetic acid (v/v) and 2 mM ammonium acetate	Positive	0.2 kV, 40 V	120 °C, 350 °C	50 L/h, 600 L/h (N_2)	Argon
Tocopherols	BEH C8	A: H_2O containing 0.02% formic acid (v/v) and 0.1 mM ammonium formate<br>B: Methanol containing 0.02% formic acid (v/v) and 0.1 mM ammonium formate	Positive	0.5 kV, 40 V	120 °C, 350 °C	50 L/h, 600 L/h (N_2)	Argon
Isoflavones	BEH C18	A: H_2O containing 0.1% formic acid (v/v)<br>B: Methanol	Positive	0.2 kV, 40 V	120 °C, 350 °C	50 L/h, 600 L/h (N_2)	Argon

Table S4. Gross Composition of SSBM and MSBM Samples

#	Sample type	Year	Crude protein (% DM)	Ash (% DM)	Ether extract (% DM)	Crude fiber (% DM)	Moisture (%)	Water activity
1	SSBM	2020	52.29	7.03	1.46	3.91	10.30	0.59
2	SSBM	2020	51.94	6.80	1.17	4.17	10.93	0.63
3	SSBM	2020	52.19	6.69	0.96	4.00	12.23	0.68
4	SSBM	2020	52.92	6.65	1.03	4.08	10.25	0.61
5	SSBM	2020	52.56	6.91	0.99	3.60	10.75	0.64
6	SSBM	2020	52.35	7.07	1.55	3.68	10.52	0.63
7	SSBM	2020	52.38	7.06	0.81	3.13	10.42	0.56
8	SSBM	2020	53.04	7.68	0.92	3.64	11.71	0.66
9	SSBM	2020	53.42	6.79	0.59	3.72	10.70	0.62
10	SSBM	2020	52.96	6.97	1.34	3.55	10.03	0.58
11	SSBM	2020	50.89	7.11	1.03	3.99	9.05	0.57
12	SSBM	2020	53.50	6.77	1.25	3.41	10.43	0.61
13	SSBM	2020	53.99	6.92	0.71	3.27	9.44	0.58
14	SSBM	2020	51.23	6.94	1.27	4.91	10.38	0.63
15	SSBM	2020	54.24	6.97	0.55	3.27	11.25	0.66
16	SSBM	2020	51.28	6.92	1.70	3.92	10.51	0.62
17	SSBM	2020	51.76	6.94	0.95	4.35	10.35	0.64
18	SSBM	2020	51.12	6.93	1.13	4.13	10.87	0.65
19	SSBM	2020	51.82	6.77	2.22	3.67	9.88	0.61
20	SSBM	2020	52.53	8.99	1.96	4.31	10.02	0.62
21	SSBM	2020	53.10	7.39	1.55	3.76	10.79	0.64
22	SSBM	2020	53.05	6.87	1.45	3.57	9.96	0.61
23	SSBM	2020	53.79	7.93	2.07	3.33	10.44	0.63
24	SSBM	2020	53.85	6.95	0.65	4.02	11.11	0.65
25	SSBM	2020	53.25	7.11	1.15	3.48	10.41	0.63
26	SSBM	2020	51.51	6.90	1.54	4.13	8.55	0.61
27	SSBM	2020	50.95	7.09	0.80	4.31	9.08	0.63
28	SSBM	2020	51.62	6.88	1.47	4.10	9.92	0.65
29	SSBM	2020	52.51	7.48	2.48	4.30	10.21	0.65
30	SSBM	2020	52.06	6.68	2.66	3.55	10.03	0.63
31	SSBM	2020	51.90	6.98	1.44	4.00	10.36	0.65
32	SSBM	2020	52.30	6.58	2.53	3.82	10.04	0.63
33	SSBM	2020	51.45	6.89	3.60	3.39	9.39	0.63

34	SSBM	2020	51.27	6.82	2.44	4.32	9.87	0.61
35	SSBM	2020	50.55	6.86	1.31	4.59	9.90	0.62
36	SSBM	2020	52.05	6.53	1.78	4.45	10.53	0.64
37	SSBM	2020	49.58	7.19	1.67	5.10	10.73	0.66
38	SSBM	2020	52.37	7.10	1.35	3.71	9.88	0.61
39	SSBM	2021	50.36	7.16	1.40	4.29	9.35	0.61
40	SSBM	2021	54.15	7.11	2.21	3.27	9.48	0.61
41	SSBM	2021	54.49	7.12	2.28	2.94	9.30	0.61
42	SSBM	2021	50.74	6.78	1.02	4.24	9.03	0.59
43	SSBM	2021	52.16	6.79	0.97	3.57	9.45	0.58
44	SSBM	2021	51.03	7.31	1.62	3.72	8.78	0.59
45	SSBM	2021	51.94	6.75	2.63	4.11	9.43	0.60
46	SSBM	2021	52.59	6.90	1.66	4.09	9.77	0.61
47	SSBM	2021	52.80	6.69	1.59	3.51	10.60	0.64
48	SSBM	2021	53.13	6.97	1.44	3.57	9.53	0.61
49	SSBM	2021	51.00	6.85	3.88	4.08	9.36	0.62
50	SSBM	2021	53.22	6.58	2.43	3.49	9.47	0.61
51	SSBM	2021	52.26	7.10	2.32	4.31	11.08	0.67
52	SSBM	2021	52.70	6.88	1.77	3.58	8.90	0.62
53	SSBM	2021	51.37	7.26	1.88	3.27	11.81	0.66
54 (HP300)*	SSBM	2021	59.22	7.44	2.19	5.00	6.97	0.37
55	MSBM	2021	46.07	6.45	5.89	6.11	5.30	0.34
56	MSBM	2021	47.54	6.07	6.86	6.68	5.24	0.40
57	MSBM	2021	44.19	6.06	6.40	5.96	5.13	0.34
58	MSBM	2021	48.52	6.19	6.58	9.06	3.71	0.30
59	MSBM	2021	45.35	6.17	6.76	6.16	4.92	0.32
60	MSBM	2021	49.23	6.08	8.93	5.67	5.53	0.40
61	MSBM	2021	48.22	6.38	6.54	5.42	4.68	0.28
62	MSBM	2021	46.07	6.39	7.41	5.96	6.83	0.45

* This sample was produced by treating conventional SSBM with a mixture of enzymes and posttreatment heating.

Table S5. Protein and Lipid Oxidation of SSBM and MSBM Samples

#	Sample type	Year	Carbonyl content (μmol/g protein)	*p*-AV	2-hexenal	2,4-heptadienal	2-heptenal	Octanal	2-octenal	Nonanal	2-nonenal	2,4-nonadienal	2-decenal	Total
					ng/g, DM									
1	SSBM	2020	5.5	0.05	207.1	621.0	5081.2	103.2	1343.7	120.0	101.9	13.8	3026.4	10618.3
2	SSBM	2020	6.8	0.54	252.5	832.1	5144.4	52.9	984.0	88.5	97.0	6.0	2904.6	10362.0
3	SSBM	2020	4.2	0.41	26.2	132.6	1490.4	15.5	137.7	38.7	12.2	4.2	559.5	2417.0
4	SSBM	2020	4.8	0.67	16.9	92.4	1323.3	3.9	51.9	35.3	6.8	2.9	321.9	1855.3
5	SSBM	2020	4.0	0.54	102.5	391.4	2982.3	36.1	539.3	47.3	43.8	3.5	1582.7	5729.0
6	SSBM	2020	6.0	0.30	5.1	14.2	421.5	4.0	14.7	35.6	7.3	4.6	59.8	566.9
7	SSBM	2020	5.7	0.67	18.0	71.7	1086.2	3.6	53.5	13.2	3.6	1.7	257.4	1509.0
8	SSBM	2020	4.2	0.94	13.8	44.1	956.7	0.0	10.4	51.1	5.8	4.2	129.2	1215.1
9	SSBM	2020	6.4	0.00	4.6	8.5	247.7	4.0	12.4	25.9	10.0	2.8	51.1	366.9
10	SSBM	2020	4.2	0.16	3.6	7.5	194.9	1.5	17.4	14.1	2.6	1.2	23.1	265.9
11	SSBM	2020	4.6	0.52	3.4	12.4	318.1	1.1	13.4	16.1	4.9	2.8	40.8	413.0
12	SSBM	2020	4.6	0.37	2.2	6.2	157.1	0.9	8.1	17.8	2.6	2.1	22.7	219.7
13	SSBM	2020	5.2	0.16	6.2	26.8	569.4	4.4	26.3	38.9	5.9	5.7	83.0	766.6
14	SSBM	2020	4.8	0.13	6.1	24.5	584.6	9.4	42.4	57.2	8.3	6.8	116.3	855.6
15	SSBM	2020	4.1	0.32	8.6	29.0	627.4	0.0	11.1	22.6	3.8	2.3	72.4	777.1
16	SSBM	2020	9.3	0.21	3.7	16.8	419.8	3.5	23.7	37.4	7.9	5.8	63.4	581.9
17	SSBM	2020	5.8	0.25	5.8	22.2	520.6	3.4	28.1	33.3	6.8	6.7	87.3	714.1
18	SSBM	2020	4.3	0.16	4.1	18.0	414.9	2.9	30.4	40.1	10.2	5.1	56.7	582.6
19	SSBM	2020	4.3	0.12	3.1	22.1	466.5	0.0	9.5	25.5	5.5	2.5	45.4	580.1
20	SSBM	2020	4.9	2.13	69.4	389.1	3475.6	5.3	166.6	47.5	15.6	1.8	1129.7	5300.5
21	SSBM	2020	6.2	0.79	24.5	57.0	1101.4	0.0	13.0	48.9	7.5	2.7	183.5	1438.5
22	SSBM	2020	5.0	0.59	3.1	12.8	303.0	3.5	18.3	46.9	10.2	6.8	50.9	455.5
23	SSBM	2020	6.6	0.60	10.9	31.4	511.6	4.4	40.0	23.5	11.2	20.1	81.0	734.2
24	SSBM	2020	6.3	0.00	4.6	12.9	334.7	3.3	18.1	35.0	9.9	5.5	49.6	473.6
25	SSBM	2020	3.1	0.15	3.1	12.1	348.0	4.5	20.4	49.4	7.2	5.6	68.8	519.1
26	SSBM	2020	4.2	0.28	7.0	26.4	650.1	5.0	36.9	44.4	11.4	7.1	120.7	909.0
27	SSBM	2020	3.6	0.08	16.0	38.8	766.5	3.5	30.7	37.0	9.2	10.7	83.5	996.0
28	SSBM	2020	4.6	0.18	12.7	20.3	472.2	7.0	28.3	107.9	17.0	17.3	69.5	752.3
29	SSBM	2020	5.3	0.96	170.9	630.6	5326.0	8.3	252.4	50.1	21.5	4.1	1428.6	7892.4
30	SSBM	2020	6.2	0.14	0.6	3.1	66.0	11.1	9.7	71.8	9.9	2.1	278.9	453.2
31	SSBM	2020	5.6	0.31	3.2	12.6	348.8	0.7	19.5	36.3	7.9	5.9	55.8	490.8
32	SSBM	2020	3.0	0.31	4.2	21.6	285.4	2.7	19.3	107.8	13.5	8.4	46.7	509.6

33	SSBM	2020	5.2	0.88	3.4	14.6	366.1	0.0	13.7	60.9	17.9	4.8	43.3	524.7
34	SSBM	2020	5.9	0.60	13.1	26.3	799.6	0.0	21.7	61.3	6.4	4.4	90.3	1023.1
35	SSBM	2020	3.8	0.23	14.2	50.0	1137.3	0.0	26.6	60.7	11.6	5.4	123.8	1429.6
36	SSBM	2020	4.1	0.56	4.4	37.8	820.4	2.2	19.1	57.0	7.0	9.6	84.8	1042.3
37	SSBM	2020	4.6	0.05	16.2	60.3	1132.9	0.0	2.1	45.6	3.1	1.0	160.9	1422.1
38	SSBM	2020	3.6	0.83	5.9	24.7	702.1	0.3	28.0	74.2	6.5	3.7	110.2	955.6
39	SSBM	2021	5.6	0.17	71.4	516.1	1291.8	0.0	130.6	0	0.6	7.0	250.4	2267.9
40	SSBM	2021	8.8	0.36	16.0	239.8	1299.1	0.0	70.3	0	2.4	0.3	198.8	1826.7
41	SSBM	2021	6.4	0.00	13.6	225.8	1351.3	0.0	57.0	0	2.6	0.0	245.0	1895.5
42	SSBM	2021	7.1	0.21	31.0	636.5	1426.0	0.0	260.9	0	11.7	4.1	835.2	3205.4
43	SSBM	2021	6.4	0.64	19.8	482.6	1108.0	0.0	122.1	0	1.7	2.5	250.8	1987.4
44	SSBM	2021	7.8	0.38	38.6	854.1	1720.3	0.0	238.5	0	7.8	6.3	593.0	3458.7
45	SSBM	2021	6.6	0.00	23.8	974.2	2431.8	0.0	212.6	0	15.8	5.8	1047.3	4711.3
46	SSBM	2021	7.7	0.58	53.8	863.6	1745.0	0.0	241.1	0	5.9	5.0	470.8	3385.2
47	SSBM	2021	7.2	0.94	30.2	725.5	1830.1	0.0	323.2	0	17.2	6.7	933.3	3866.1
48	SSBM	2021	4.5	0.28	10.9	228.0	990.4	43.0	145.0	0	141.8	2.4	1876.1	3437.7
49	SSBM	2021	9.4	0.98	18.8	459.6	2733.0	0.0	378.1	0	26.8	16.7	1076.2	4709.2
50	SSBM	2021	5.9	0.69	3.5	56.3	788.0	1.2	140.7	0	15.4	7.8	181.7	1194.6
51	SSBM	2021	4.4	0.53	12.3	242.1	1386.8	0.0	113.8	0	4.5	7.1	249.4	2016.0
52	SSBM	2021	7.8	0.02	6.8	89.3	1050.2	0.0	215.2	0	5.4	8.7	274.7	1650.4
53	SSBM	2021	6.8	0.93	4.0	32.0	426.7	11.1	40.0	39.3	2.0	4.3	133.8	693.3
54 (HP300) *	SSBM	2021	9.3	2.29	75.8	118.2	1779.2	12.1	148.6	21.8	11.2	3.5	423.6	2594.0
55	MSBM	2021	7.7	0.79	9.8	33.7	309.4	24.7	52.8	96.4	2.8	4.6	105.6	639.9
56	MSBM	2021	10.9	1.58	29.1	88.4	759.0	25.7	102.3	106.2	6.8	6.7	246.4	1370.6
57	MSBM	2021	11.4	0.88	26.4	88.1	1780.8	27.8	142.4	87.1	22.0	7.2	457.5	2639.2
58	MSBM	2021	14.1	0.49	629.1	1791.7	17492.8	161.4	2364.8	215.4	227.8	21.2	9671.3	32575.6
59	MSBM	2021	6.4	1.31	8.9	42.2	619.1	14.5	50.3	57.8	4.2	6.2	205.3	1008.5
60	MSBM	2021	5.2	1.73	35.4	122.4	1241.7	30.0	159.9	97.0	8.2	6.7	360.8	2062.1
61	MSBM	2021	8.3	1.49	10.3	52.9	174.1	15.4	27.4	64.0	4.2	12.4	35.8	396.5
62	MSBM	2021	9.7	1.71	160.9	456.6	5048.6	120.9	1009.7	172.8	59.6	25.8	1884.0	8938.8

* This sample was produced by treating conventional SSBM with a mixture of enzymes and posttreatment heating.

Table S6. Antioxidants of SSBM and MSBM Samples

#	Sample type	Year	TEAC	Total phenolic content (mg/g caffeic acid equivalent DM)	α-tocopherol	γ-tocopherol	Daidzin	Genistin	Daidzein	Genistein
					μg/g DM					
1	SSBM	2020	24.6	1.05	0.008	6.1	397.8	1527.6	42.3	42.3
2	SSBM	2020	25.0	1.34	0.015	5.7	577.1	2153.4	42.7	35.9
3	SSBM	2020	25.0	1.34	0.003	5.9	451.2	1488.0	14.8	17.1
4	SSBM	2020	25.4	1.14	0.008	5.6	460.2	1551.0	21.2	20.1
5	SSBM	2020	24.3	1.32	0.001	1.7	384.3	1166.4	15.7	15.7
6	SSBM	2020	25.9	1.24	0.001	5.9	516.3	1865.2	35.8	34.6
7	SSBM	2020	24.6	1.14	0.010	1.6	428.7	1444.5	20.1	20.1
8	SSBM	2020	25.5	1.25	0.017	4.4	427.0	1500.7	61.2	48.7
9	SSBM	2020	25.1	1.11	0.015	7.8	486.0	1753.6	34.7	33.6
10	SSBM	2020	25.3	1.16	0.000	7.3	436.8	1480.5	16.7	17.8
11	SSBM	2020	25.7	1.32	0.001	6.3	469.5	1655.9	20.9	22.0
12	SSBM	2020	24.4	1.37	0.004	7.0	470.0	1558.6	24.6	24.6
13	SSBM	2020	25.0	1.27	0.002	2.4	491.4	1866.2	23.2	27.6
14	SSBM	2020	25.0	1.23	0.003	6.7	421.8	1418.2	17.9	17.9
15	SSBM	2020	24.8	1.12	0.006	4.1	442.8	1542.5	45.1	39.4
16	SSBM	2020	25.0	1.09	0.002	7.8	539.7	1891.8	31.3	30.2
17	SSBM	2020	24.6	1.17	0.005	8.3	441.7	1459.0	15.6	16.7
18	SSBM	2020	25.2	1.29	0.003	6.7	535.2	1807.5	26.9	25.8
19	SSBM	2020	25.3	1.23	0.004	13.1	512.6	1643.4	24.4	23.3
20	SSBM	2020	25.3	1.22	0.004	8.9	445.7	1469.2	43.3	35.6
21	SSBM	2020	25.5	1.19	0.007	8.8	483.1	1705.0	41.5	34.7
22	SSBM	2020	24.6	1.36	0.007	10.2	459.8	1542.6	22.2	22.2
23	SSBM	2020	25.2	1.17	0.001	8.5	413.1	1545.3	40.2	38.0
24	SSBM	2020	25.5	1.15	0.011	9.3	495.0	1825.9	33.7	31.5
25	SSBM	2020	25.0	1.03	0.012	7.3	456.5	1593.9	26.8	24.6
26	SSBM	2020	24.2	1.08	0.003	5.7	444.0	1515.6	23.0	21.9
27	SSBM	2020	24.0	1.08	0.009	1.8	414.7	1377.0	18.7	16.5
28	SSBM	2020	24.5	1.20	0.002	25.9	439.6	1452.0	12.2	13.3
29	SSBM	2020	24.5	1.06	0.001	1.6	291.8	1114.8	21.2	20.0
30	SSBM	2020	25.2	0.72	0.059	3.1	619.1	2198.5	30.0	34.5
31	SSBM	2020	24.9	1.13	0.012	8.0	387.1	1327.5	12.3	13.4
32	SSBM	2020	24.8	1.17	0.012	9.1	499.1	1788.6	34.5	30.0
33	SSBM	2020	25.2	1.16	0.004	20.0	512.1	1842.0	35.3	32.0

34	SSBM	2020	24.9	1.24	0.001	6.6	482.6	1690.9	15.5	17.8
35	SSBM	2020	25.1	0.98	0.007	4.5	581.6	2105.4	23.3	25.5
36	SSBM	2020	25.1	1.12	0.003	7.0	477.3	1542.4	20.1	19.0
37	SSBM	2020	25.7	1.10	0.002	5.8	506.3	1802.4	25.8	23.5
38	SSBM	2020	24.7	1.10	0.001	5.5	436.1	1495.8	21.1	18.9
39	SSBM	2021	22.6	0.96	0.011	3.4	453.4	1533.4	23.2	19.9
40	SSBM	2021	23.9	0.64	0.012	4.4	449.6	1472.6	32.0	26.5
41	SSBM	2021	24.1	1.01	0.001	4.3	458.7	1637.3	39.7	34.2
42	SSBM	2021	25.9	1.22	0.002	5.6	536.4	1931.4	35.2	29.7
43	SSBM	2021	27.2	1.33	0.001	4.1	522.4	1782.4	37.5	30.9
44	SSBM	2021	26.9	1.48	0.013	8.3	535.0	1839.5	36.2	31.8
45	SSBM	2021	26.2	1.23	0.007	17.8	403.0	1330.5	33.1	27.6
46	SSBM	2021	25.0	1.07	0.034	10.5	410.1	1546.0	27.7	26.6
47	SSBM	2021	24.7	1.07	0.005	7.9	415.0	1550.3	36.9	35.8
48	SSBM	2021	22.3	0.96	0.004	3.8	392.4	1404.9	22.1	23.2
49	SSBM	2021	24.6	1.15	0.012	5.2	417.0	1523.6	15.4	27.6
50	SSBM	2021	26.0	1.24	0.010	16.8	417.5	1602.8	34.2	29.8
51	SSBM	2021	26.4	1.11	0.009	8.6	376.7	1368.6	30.4	23.6
52	SSBM	2021	25.6	1.22	0.002	1.0	355.7	1182.2	29.6	13.2
53	SSBM	2021	23.7	1.31	0.003	9.8	538.6	1680.5	40.8	36.3
54 (HP300) *	SSBM	2021	26.9	0.97	0.011	6.9	402.0	1384.5	113.9	66.6
55	MSBM	2021	23.3	0.68	0.011	9.7	264.0	861.7	21.1	31.7
56	MSBM	2021	22.6	0.56	0.014	23.1	395.7	1240.0	30.6	34.8
57	MSBM	2021	24.2	0.93	0.137	21.8	375.3	1222.7	56.9	56.9
58	MSBM	2021	23.8	0.54	0.560	16.2	300.1	1082.1	48.8	43.6
59	MSBM	2021	23.0	0.95	0.018	15.1	253.5	726.8	16.8	18.9
60	MSBM	2021	23.3	0.91	0.004	8.7	347.2	899.8	23.3	23.3
61	MSBM	2021	24.9	1.20	0.046	9.0	411.2	1377.5	35.7	39.9
62	MSBM	2021	23.8	1.12	0.018	10.3	322.0	1151.7	48.3	50.4

* This sample was produced by treating conventional SSBM with a mixture of enzymes and posttreatment heating.

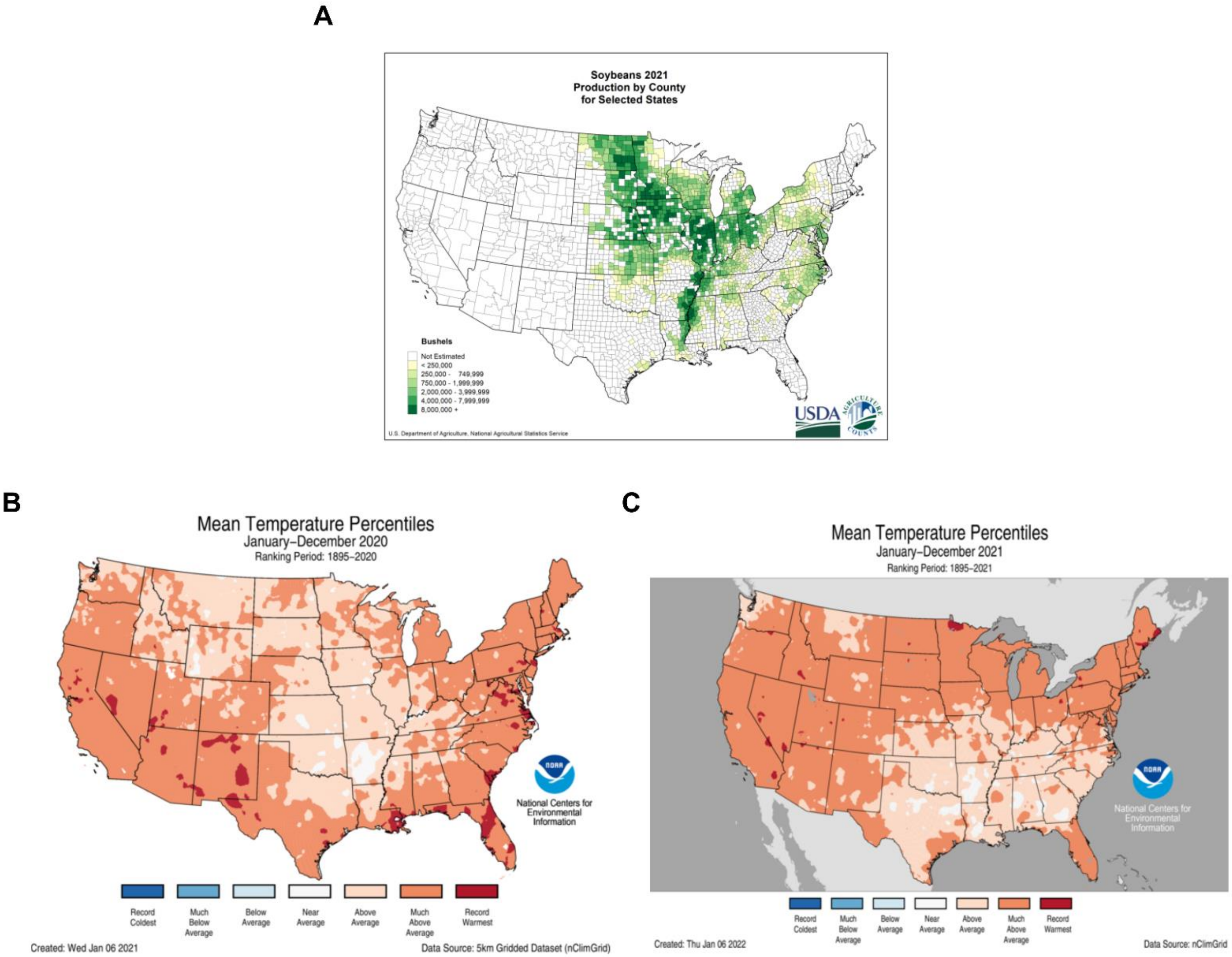

Figure S1. Soybean Production Map and Mean Temperature in the United States. (**A**) USDA map of 2021 soybeans production (copied from https://www.nass.usda.gov/Charts_and_Maps/Crops_County/sb-pr.php), **(B)** NOAA map of mean temperature percentiles in 2020 (copied from https://www.ncei.noaa.gov/news/national-climate-202012), **(C)** NOAA map of mean temperature percentiles in 2021 (copied from https://www.ncei.noaa.gov/news/national-climate-202112).
